# Identification and Functional Speculation of Genes Related to Sex Pheromone Synthesis Expressed in the Gonads of Female *Gynaephora qinghaiensis* (Lepidoptera: Lymantriidae)

**DOI:** 10.3390/genes16091046

**Published:** 2025-09-05

**Authors:** Zhanling Liu, Shujing Gao, Haibin Han, Xiaorui Wang, Guixiang Kou, Haishun Wang, Yuantao Zhou

**Affiliations:** 1 College of Agriculture and Animal Husbandry, Qinghai University, Xining 810016, China; liu20000607ling@163.com (Z.L.); 15564306290@163.com (X.W.); kgx3272647933@163.com (G.K.); 15500741771@163.com (H.W.); 2 Institute of Grassland, Chinese Academy of Agricultural Sciences, Hohhot 010010, China; 3 College of Horticulture and Plant Protection, Inner Mongolia Agricultural University, Hohhot 010018, China; hhb.25@163.com

**Keywords:** *Gynaephora qinghaiensis*, sex pheromone glands, sex pheromone biosynthesis related genes, bioinformatics analysis, expression profile analysis

## Abstract

**Background:** Grassland desertification has garnered significant attention as a pressing issue. Among the key pests affecting plateau meadows, the *Gynaephora qinghaiensis* (Lepidoptera: Lymantriidae) poses a substantial threat in the Qinghai-Tibet Plateau region, highlighting the urgent need for effective, environmentally friendly control strategies. Insect sex pheromones are increasingly employed in pest monitoring and management. **Methods:** This study aims to identify and analyze genes associated with sex pheromone synthesis in grassland caterpillars through transcriptome sequencing and tissue-specific expression analysis. **Results:** A total of 139,599 transcripts and 56,403 Unigenes were obtained from the sex pheromone glands transcriptome database. A total of 31 genes related to sex pheromone synthesis were identified, including 1 *ACC*, 8 *DES*, 6 *AR*, 7 *FAR*, 5 *FAS*, and 4 *ACT* genes. The expression levels of these genes varied significantly across different tissues in both male and female caterpillars (*p* < 0.05). *GqinACC1*, *GqinDES1*, *GqinDES4*, *GqinDES8*, *GqinAR3*, *GqinFAR6*, *GqinACT2*, and *GqinACT3* exhibited significantly higher expression levels in the female gonads compared to other tissues (*p* < 0.01). **Conclusions:** We hypothesize that specific genes play specific roles in the pheromone synthesis pathways of pests, Key genes were identified based on expression patterns for subsequent functional studies. The results of this study offer valuable data support for subsequent investigations into the mechanisms underlying sex pheromone synthesis in *G. qinghaiensis*. Additionally, these findings may identify potential targets for future research on genes associated with pheromone biosynthesis, which could disrupt their chemical communication and contribute to grassland conservation efforts.

## 1. Introduction

In recent years, disruption of pheromone-mediated mating has proven effective for pest management in species such as *Chilo suppressalis* [1], *Cnaphalocrocis medinalis* [2], and *Cossus insularis* [3]. In many moths of the order Lepidoptera, reproductive isolation is largely dependent on sexually mature females producing and releasing species-specific sex pheromone components to attract males of the same species. Adult males utilize their antennae to detect these pheromone components, allowing them to locate females successfully and complete mating behaviors [4]. Insect sex pheromones, also referred to as sex pheromones, are trace chemical signaling substances secreted by specialized glands of sexually mature individuals of a specific sex within the same species. These pheromones are released into the environment and detected by the sensory organs of individuals of the opposite sex, facilitating successful courtship and mating. Most sex pheromones are produced by females [5]. In lepidopteran female moths, the glands that produce sex pheromones are typically located in the eighth and ninth abdominal segments [6,7,8,9]. However, in some species, these glands can be found on the dorsal surface of the thorax or on the wings [10]. In Lepidoptera, male moths primarily secrete sex pheromones from their abdomens and wings [11]. In 1959, Butenandt was the first to isolate sex pheromones from the *Bombyx mori* [12]. Since then, an increasing number of insect sex pheromone components have been identified, with lepidopteran sex pheromones being the most extensively studied. Most moths release mainly C10-C18 linear unsaturated fatty acids containing oxidative functional groups such as aldehyde, alcohol, or acetate [13,14,15,16,17,18,19,20]. Various enzymes are required for pheromone biosynthesis, including acetyl-CoA carboxylase (ACC), fatty acid synthase (FAS), desaturase (DES), fatty acyl reductase (FAR), acetyltransferase (ACT), and aldehyde reductase (AR), which collectively convert fatty acid precursors into species-specific pheromone components [21,22,23,24,25]. Transcriptomic studies have successfully identified pheromone biosynthesis genes in several lepidopteran species, revealing tissue-specific expression patterns in pheromone glands that correlate with functional roles in biosynthesis [6,26,27,28,29,30]. However, the quantities of sex pheromones secreted by insects are very small, necessitating reliance on artificial synthesis for pest control applications [31]. Therefore, studying the mechanisms of sex pheromone synthesis is crucial.

*G. qinghaiensis* belongs to the order Lepidoptera and the family Lymantriidae, and is a common herbivorous pest in alpine meadows. This insect mainly feeds on high-quality forage grasses of the families Poaceae and Cyperaceae. There is a significant morphological difference between male and female adults, with the thoracic legs, wings, and antennae of female adults being degenerate [32]. *G. qinghaiensis* is a holometabolous insect and has four developmental stages: eggs, larvae, pupae and adults. The larval stage is the nutritional stage for *G. qinghaiensis* to feed, and it is also the harmful stage of heavy grazing; When insect population density is high, typically ranging from 200 to 500 individuals per square meter [33], it can lead to a shortage of food for livestock. In cases of severe damage, these infestations can alter the structure of grassland plant communities, exacerbate grassland degradation, and contribute to the deterioration of the overall ecological environment [34,35,36]. It has been reported that *G. qinghaiensis* exhibits strong reproductive capabilities. The larvae possess poison glands on their backs, which can cause allergic reactions in livestock and humans. Additionally, they have adapted to extremely harsh environments, including strong ultraviolet radiation, low oxygen levels, and severe cold, making them difficult to prevent and control [37]. Despite extensive efforts toward their management since the 1960s, the population of *G. qinghaiensis* has not been fundamentally or effectively controlled [38].

In recent years, research on sex pheromones has highlighted their significant role in the monitoring and control of insects. Successful examples of using sex pheromones for the management of lepidopteran pests, such as *Cydia nigriana* [39] and *Adoxophyes honmai* [40], provide a new approach for monitoring and controlling caterpillar populations in the grasslands of Qinghai. Currently, research on the sex pheromones of *G. qinghaiensis* mainly focuses on the crude extracts of female moth sex pheromones and the attraction of male moths to synthetic sex pheromones [41,42]. However, the active components of these sex pheromones and the mechanisms underlying their synthesis remain unclear.

As a major pest of alpine meadows, further investigation into the genes involved in sex pheromone biosynthesis may provide potential targets for disrupting chemical communication and aiding in grassland conservation. In this study, we utilized the gonadal transcriptome database of *G. qinghaiensis* to screen and identify genes associated with sex pheromone synthesis. We analyzed the physicochemical properties, phylogenetic relationships, and tissue expression profiles of these genes using bioinformatics methods and RT-qPCR technology. This research aims to establish a foundation for a more in-depth understanding of the mechanisms underlying sex pheromone synthesis in *G. qinghaiensis*, ultimately offering new strategies for pest control.

## 2. Materials and Methods

### 2.1. Insects

In June 2023, *G. qinghaiensis* larvae were collected in Haiyan County, Haibei Prefecture, Qinghai Province, and the larvae were reared in the laboratory until adult emergence. The heads, thorax, abdomens, male antennae (antennae of female adults were degenerated), ovary and sex pheromone glands of female adult worms were dissected and collected. A total of 9 samples were collected, with 3 biological replicates of each sample, each containing 10 adult tissues. The samples were labeled and immediately frozen in liquid nitrogen (Chengsheng calcium carbide supply station, Xining, China) and stored at −80 °C for later use. Note that in order to prevent decomposition and contamination of the glands during dissection, the glands were dissected on ice under a microscope (Osmicro Optical Instruments Co., Ltd., Shenzhen, China); after the head was removed with a scalpel, three or four sections of the abdomen were gently compressed to force the glands to protrude from the end of the abdomen, and the glands were cut with surgical scissors and quickly stored in liquid nitrogen [43].

### 2.2. Total RNA Extraction

Total RNA was extracted from each of the nine tissue samples using the TRIzol method. The integrity and DNA contamination of the RNA samples were evaluated via 1% agarose gel electrophoresis, whereas RNA concentration was determined using a fluorescence spectrophotometer. Additionally, the purity of RNA in each tissue sample was assessed with the same spectrophotometer(Olympic Scientific Instruments Co., Ltd., Shanghai, China). Samples with RNA purity within the acceptable range (OD_260_ nm/OD_280_ nm ratio of 1.8–2.2) were subjected to reverse transcription using the M-MLV Reverse Transcription Kit (Beijing Solarbio Science & Technology Co., Ltd., Beijing, China). The synthesized cDNA was stored at −20 °C for subsequent RT-qPCR analysis. For further validation, the integrity of RNA isolated from sex pheromone glands was accurately determined using an Agilent 2100 Bioanalyzer (Agilent Technologies Ltd., Beijing, China). The quality criteria for the extracted RNA were defined as follows: OD_260_ nm/OD_280_ nm ratio of 1.8–2.2, 28S/18S rRNA ratio > 2, OD_260_ nm/OD_230_ nm ratio > 1.8, and RNA Integrity Number (RIN) > 8.5. All these parameters confirmed that the extracted RNA was of high quality.

### 2.3. Library Construction and Quality Inspection

Once the RNA samples were deemed qualified, library construction commenced. Eukaryotic mRNA was enriched using magnetic beads with Oligo (dT). A fragmentation buffer was added to randomly fragment the mRNA. The first strand of cDNA was synthesized using the mRNA as a template, followed by the addition of the buffer, dNTPs, RNase H, and DNA polymerase I. The resulting cDNA was then purified using AMPure XP beads. The purified double-stranded cDNA underwent end repair, A-tailing, and ligation with sequencing adapters. Fragment size selection was performed using AMPure XP beads to ensure the desired insert size. Finally, the cDNA library was obtained through PCR enrichment. After library construction, quality checks were conducted. Initial quantification was performed using a Qubit 2.0, and the insert size of the library was assessed with an Agilent 2100. The insert size was confirmed to be as expected before proceeding to the next steps. The Q-PCR method was employed to accurately quantify the effective concentration of the library, ensuring it exceeded 2 nM. Once the library passed quality inspection, sequencing was performed on the Illumina NovaSeq 6000 platform (Illumina, San Diego, CA, USA), utilizing a read length of PE150. Library construction and quality inspection were assisted by Beijing Novo Zhiyuan Technology Co., Ltd. (Beijing, China), DNA fragments are fragmented to approximately 350 bp, followed by end repair, A-tailing, adapter ligation, purification, and PCR amplification. (library quality control: concentration, ≥2 nmol/L (or 2 ng/μL); volume, ≥15–100 μL depending on data volume; insert size, approximately 350 bp; total passing filter rate, ≥55%; Q30 of Read1/Read2, ≥80%).

### 2.4. Transcriptome Data Assembly and Gene Annotation

The raw reads obtained from RNA-seq data underwent quality filtering to produce clean data. Trinity software (v2.11.0) was utilized for transcriptome assembly. Initially, the sequencing reads were fragmented into shorter segments (K-mers), which were then extended into longer contiguous sequences (Contigs). By analyzing overlaps between these fragments, a set of components was generated. The De Bruijn graph method, combined with sequencing read information, was used to identify transcript sequences within each component set. To annotate the Unigene sequences, we performed comparisons against several databases, including NR, Swiss-Prot, GO, COG, KOG, and KEGG, using BLAST software (2.14.0). Additionally, the amino acid sequences of the Unigenes were predicted and subsequently compared with the Pfam database using HMMER3.2.1 (default threshold) software to obtain comprehensive Unigene annotation information.

### 2.5. Bioinformatics Analysis

In the transcriptome database of the *G. qinghaiensis* gonads, genes related to sex pheromone synthesis were screened or identified as “ACC” (Acetyl-CoA carboxylase), “ACT” (Acetyltransferase), “FAR” (fatty acyl reductase), “DES” (Desaturase), “FAS” (fatty acid synthase), and “AR” (Aldehyde reductase) [7]. Relevant gene sequences were screened and subjected to BLASTn comparisons on the NCBI website, with a similarity threshold set to greater than 60%. The open reading frames (ORFs) were predicted using the online tool ORFfinder (https://www.ncbi.nlm.nih.gov/orffinder/ (accessed on 26 December 2023)). Additionally, the online tool ProtParam (https://web.expasy.org/protparam/ (accessed on 10 July 2025)) was employed to perform bioinformatics analyses on the identified genes. Using Signalp 4.1 Server (http://cbs.dtu.dk/services/Signalp/ (accessed on 28 December 2023)) to predict its signal peptide, MEGA7.0 was used to construct the phylogenetic tree. The amino acid sequences were aligned with ClustalW (1000 iterations of super fast self-expansion); maximum-likelihood trees of Des and FAR were constructed by the IQTree program using the LG + I + G4 and LG + F + R6 models, respectively, implemented with the default settings and 1000 ultrafast bootstrap approximation.

### 2.6. RT-qPCR Detection of Target Genes

The relative expression levels of each gene were based on the internal reference gene (RPS15). Using synthetic cDNA samples (head, thorax, abdomen, antennae, ovary, and sex pheromone glands of female adults) as templates. Amplification system (20 μL): positive and negative primers, 0.8 μL; Master Mix, 10 μL; ddH_2_O, 7.4 μL; and cDNA template, 1 μL. The reaction was performed using a real-time fluorescence quantitative PCR instrument (ABI7500 type). The PCR amplification conditions were as follows: initial denaturation at 95 °C for 1 min; denaturation at 95 °C for 15 s; and annealing at 60 °C for 15 s and, 72 °C for 45 s for, 40 cycles. The expression levels in the antennae of male adult worms were compared. Three biological replicates and negative control groups (no template)were set up for each sample. To calculate relative levels of expression, we followed the comparative 2^−ΔΔCt^ method (amplification efficiency of all genes was recorded as almost to 100%).

### 2.7. Primer Design and Synthesis

Thirty-one gene sequences (1 ACC, 8 DES, 6 AR, 7 FAR, 5 FAS, and 4 ACT) were selected and identified from the sex pheromone glands transcriptomes database of female adult *G. qinghaiensis*. Primers were designed using Oligo7.60 software and synthesized by Beijing Ruboxingke Biotechnology Co., LTD. (Beijing, China). The reference gene (Ribosomal protein S15) primer was derived from the Insect Laboratory of Lanzhou University [44] (Table S1).

### 2.8. Data Analysis

The relative expression levels of genes related to sex pheromone synthesis in different tissues of *G. qinghaiensis* were calculated using the 2^−ΔΔCt^ value method, compared with the expression levels in male antennae. The data were analyzed by one-way analysis of variance (ANOVA) and significance difference test (LDS) using SPSS 21.0 software [6]. At the same time, the software Graphpad Prism 10 was used for the *t*-test (*p* < 0.05), and the tissue expression profile was drawn. The expression difference between males and females was compared by Duncan’s new complex range method.

## 3. Results

### 3.1. Transcriptome Sequence Assembly

Transcriptome sequencing of three sex pheromone gland samples (three biological replicates) from the *G. qinghaiensis* was conducted using the Illumina HiSeq™ 2000 high-throughput sequencing platform. A total of 56,403 Unigenes were annotated, yielding an aggregate base count of 7.6 Gb. The quality metrics indicated robust sequencing data: Q20 was 98.60%, while the Q_30_ value (the percentage of bases with a quality score ≥ 30) was 96.26%. Additionally, the GC content was determined to be 39.51% (all the data above are averages) (Table 1). These results demonstrate that the sequencing data quality is high, providing a reliable foundation for subsequent data analysis.

A total of 139,599 transcripts were generated following the assembly process, resulting in the identification of 56,403 Unigenes. Among these, 22,694 unigenes fell within the length range of 300–500 bp, 16,624 genes were categorized in the 500–1000 bp range, 9273 genes were between 1000 and 2000 bp, and 7812 genes exceeded 2000 bp. This indicates a high level of assembly integrity (Table 2), confirming the data’s suitability for subsequent biological analyses.

### 3.2. Unigene Function Annotation

The gonadal transcriptomes of *G. qinghaiensis* were annotated across seven databases: COG, GO, KEGG, KOG, Pfam, Swiss-Prot, and NR. The analysis revealed that the KEGG database contained the highest number of annotated genes, with 20,576 entries (36.48%), followed by NR with 13,781 entries (24.43%). Other annotations included Pfam (11,603 genes, 20.57%), Swiss-Prot (11,001 genes, 19.50%), KOG (10,721 genes, 19.01%), GO (10,228 genes, 18.13%), and the COG database, which had the fewest annotations with 4865 genes (8.6%) (Table 3).

### 3.3. Bioinformatics Analysis

In this study, a total of 31 genes related to sex pheromone synthesis were identified in the gonadal transcriptome database of *G. qinghaiensis*. (Table 2). ORFfinder and NCBI BLASTp analysis revealed that the nucleotide length of the target gene was 381–1602 bp. TMHMM2.0 was used to predict its transmembrane domain. The analysis of the sex pheromone synthesis genes revealed that out of 31 genes examined, 13 exhibited between 1 to 5 transmembrane domains, indicating potential roles in membrane-associated processes. Notably, *GqinFAR2* showed a high sequence similarity of 96.88% with the gene from *Streltzoviella insularis* (GenBank entry QLI61998.1), suggesting a close evolutionary relationship. Similarly, *GqinFAS4* displayed a similarity of 87.23% with the *Spodoptera litura* gene (GenBank entry XP_022831505.1), while *GqinDES2* showed an 86.60% similarity with the *Spodoptera exigua* gene (GenBank entry ARD71182.1). Additionally, the similarity between the other target genes and genes from various lepidopteran species was consistently above 50%, indicating a significant level of conservation among these genes across different species. This information may provide insights into the functional roles of these genes in pheromone synthesis and their evolutionary significance. These results indicate that the sex pheromone-related genes of the *G. qinghaiensis* may be homologous to those in other Lepidoptera (Table 4).

### 3.4. Physicochemical Properties of Genes

The prediction of physical and chemical properties of the genes related to sex pheromone synthesis of the *G. qinghaiensis* showed that the amino acid length was between 127 and 533 aa and the relative molecular weight was between 14.03 and 60.50 kDa. The aliphatic index is high, between 63.78 and 107.52, which belongs to the heat -stable protein. The isoelectric points of *GqinACC1*, *GqinAR2*, *GqinAR4*, *GqinFAR2*, *GqinFAS1* and *GqinACT1*-*2* were between 4 and 6 (acidic protein). The isoelectric points of *GqinDES2*-*6*, *GqinDES8*, *GqinAR3*, *GqinAR5-6*, *GqinFAR4-7*, *GqinFAS4* and *GqinACT4* were between 8 and 10 (partial basic protein). The isoelectric points of *GqinDES1*, *GqinDES7*, *GqinAR1*, *GqinFAR3*, *GqinFAS2*, *GqinFAS5*, and *GqinACT3* were between 6 and 7 (weakly acidic protein). The positive and negative residues of the target gene ranged from 11 to 64 and 16 to 63 respectively. The instability index of *GqinACC1*, *GqinDES1*, *GqinDES6*, *GqinAR6*, *GqinFAR5*-*6*, *GqinFAS1*-*2*, and *GqinACT2* was greater than 40 (unstable protein properties). The average hydropathicity of *GqinACC1* and *GqinACT1* was < −0.5 (hydrophilic protein). The average hydropathicity of the remaining genes was between −0.5 and 0.5 (amphoteric proteins) (Table 5).

### 3.5. Phylogenetic Analysis

**Figure 1:**
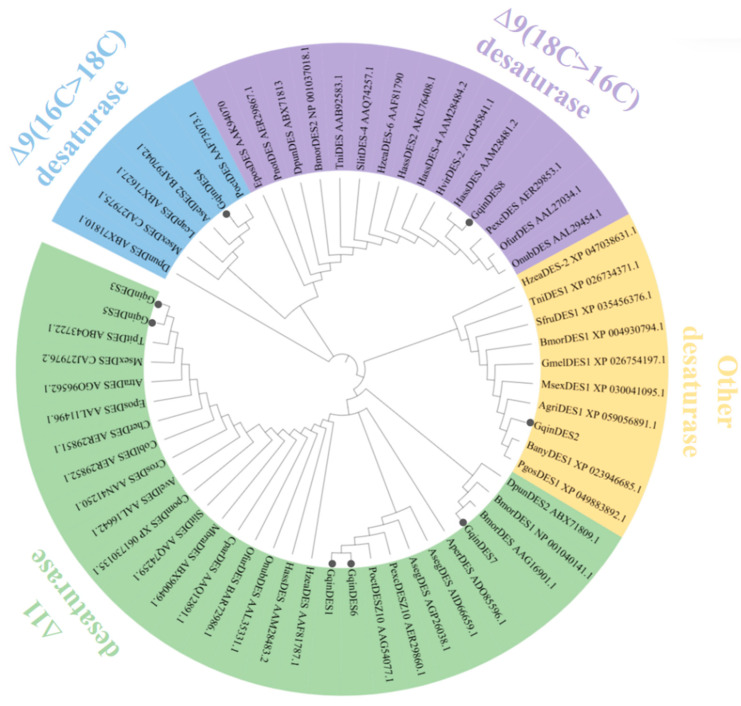
Phylogenetic tree of GqinDES and other identified Lepidopteran DESs. *A. grisella* (Agri), *A. segetum* (Aseg), *Amyelois transitella* (Atra), *Bicyclus anynana* (Bany), *B. mori* (Bmor), *Cydia pomonella* (Cpom), *Dendrolimus punctatus* (Dpun), *Galleria mellonella* (Gmel), *H. assulta* (Hass), *Helicoverpa zea* (Hzea), *Heliothis virescens* (Hvir), *Manduca sexta* (Msex), *Melitaea cinxia* (Mcin), *Spodoptera frugiperda* (Sfru), *S. litura* (Slit), *T. ni* (Tni), *Lampronia capitella* (Lcap), *Ascotis selenaria* (Asel), *Planotortrix octo* (Poct), *E. postvittana* (Epos), *Planotortrix notophaea* (Pnot), *Planotortrix excessana* (Pexc), *O. furnacalis* (Ofur), *Ostrinia nubilalis* (Onub), *Antheraea pernyi* (Aper), *Choristoneura parallela* (Cpar), *Mamestra brassicae* (Mbra), *Argyrotaenia velutinana* (Avel), *Choristoneura rosaceana* (Cros), *Ctenopseustis obliquana* (Cobl), *Ctenopseustis herana* (Cher), *Thaumetopoea pityocampa* (Tpit). *G. qinghaiensis* isrepresented by black circles.

**Figure 2:**
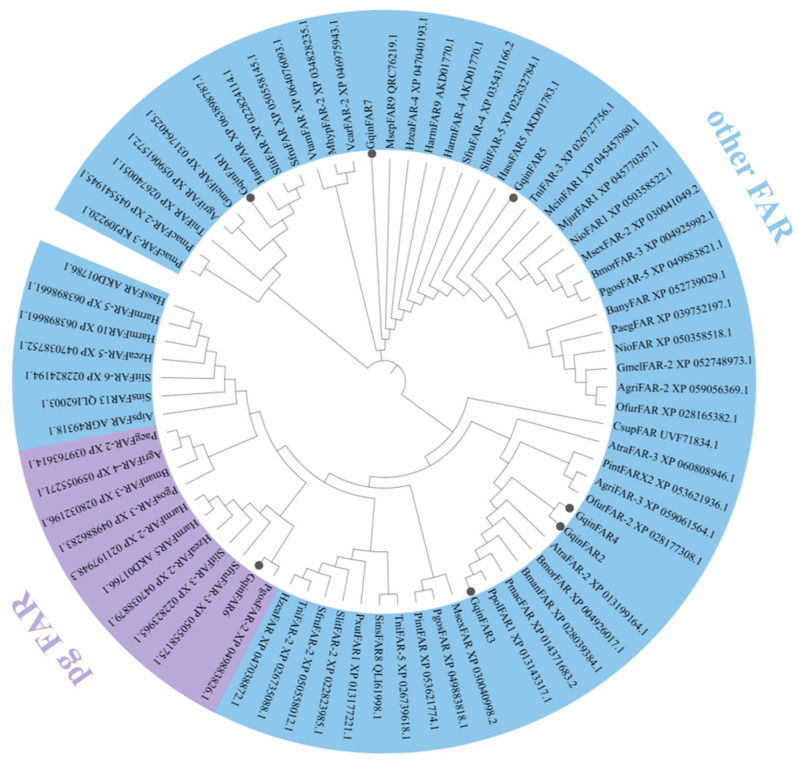
Phylogenetic tree of GqinFAR and other identified Lepidopteran FARs. *A. grisella* (Agri), *Agrotis ipsilon* (Aips), *A. transitella* (Atra), *B. anynana* (Bany), *Bombyx mandarina* (Bman), *B. mori* (Bmor), *C. suppressalis* (Csup), *G. mellonella* (Gmel), *H. armigera* (Harm), *H. assulta* (Hass), *Helicoverpa zea* (Hzea), *M. sexta* (Msex), *Maniola hyperantus* (Mhyp), *Maniola jurtina* (Mjur), *M. cinxia* (Mcin), *Mythimna separata* (Msep), *Nymphalis io* (Nio), *O. furnacalis* (Ofur), *Papilio machaon* (Pmac), *Papilio polytes* (Ppol), *Papilio xuthus* (Pxut), *Pararge aegeria* (Paeg), *P. gossypiella* (Pgos), *P. interpunctella* (Pint), *Spodoptera frugiperda* (Sfru), *S. litura* (Slit), *S. insularis* (Sins), *T. ni* (Tni), *Vanessa cardui* (Vcar), *Vanessa tameamea* (Vtam). *G. qinghaiensis* is represented by black circles.

Phylogenetic analysis was conducted to explore the evolutionary relationships between the identified GqinDES sequences and other desaturase protein sequences from lepidopteran species. A phylogenetic tree was constructed using appropriate methods, such as neighbor-joining, based on the amino acid sequences of the desaturases. The analysis revealed that the eight GqinDES sequences were distributed across various desaturase branches in insects. Specifically, GqinDES1 and GqinDES6 were found to cluster together, indicating a close evolutionary relationship. Meanwhile, GqinDES3, GqinDES5, and GqinDES7 were grouped within the ∆11 desaturase branch, suggesting a shared functional role in pheromone biosynthesis. Additionally, GqinDES4 was classified into the ∆9 (16C > 18C) desaturase homologous clade, and GqinDES8 was positioned within the ∆9 (18C > 16C) desaturase branch. In contrast, GqinDES2 exhibited a broader clustering pattern with other desaturases, indicating potential functional diversity (Figure 1). The GqinFARs phylogenetic analysis revealed that GqinFAR5 and GqinFAR7 were retained in the same clade. In contrast, GqinFAR3 and GqinFAR6 clustered together with other homologous species from lepidopterans. Additionally, GqinFAR2 and GqinFAR4 formed a separate group, clustering alongside the *Achroia grisella*, *O. furnacalis*, and *Plodia interpunctella*. It is noteworthy that among the seven GqinFARs, only GqinFAR6 was classified within a clade of lepidopteran pgFARs. The remaining GqinFARs were found to be closely related to these insect FARs, suggesting similar functional roles (Figure 2).

These findings contribute to our understanding of the evolutionary dynamics of desaturase genes and fatty acyl-CoA reductase in lepidopteran species and may provide insights into their roles in sex pheromone synthesis.

### 3.6. Analysis of Tissue Expression Profiles

In this study, RT-qPCR was used to study the expression of genes related to sex pheromone synthesis in different tissues (head, thorax, abdomen, antennae, ovary, and sex pheromone glands) of *G. qinghaiensis* by using the expression levels of each gene in male and female antennae as a control. The results showed that the expression levels of target genes in different tissues of male and female caterpillars were different. A total of 31 genes associated with sex pheromone synthesis were expressed in both PG and male antennae. Notably, *GqinACC1*, *GqinDES1*, *GqinDES4*, *GqinDES8*, *GqinAR3*, *GqinFAR6*, *GqinACT2* and *GqinACT3* were significantly expressed in PG. Among these, *GqinDES1* and *GqinFAS5* displayed the highest expression levels in the PG (*p* < 0.01), with *GqinDES1* being approximately 100 times more highly expressed than other genes, and GqinFAS5 showing around 1000 times higher expression compared to the rest.

**Figure 3:**
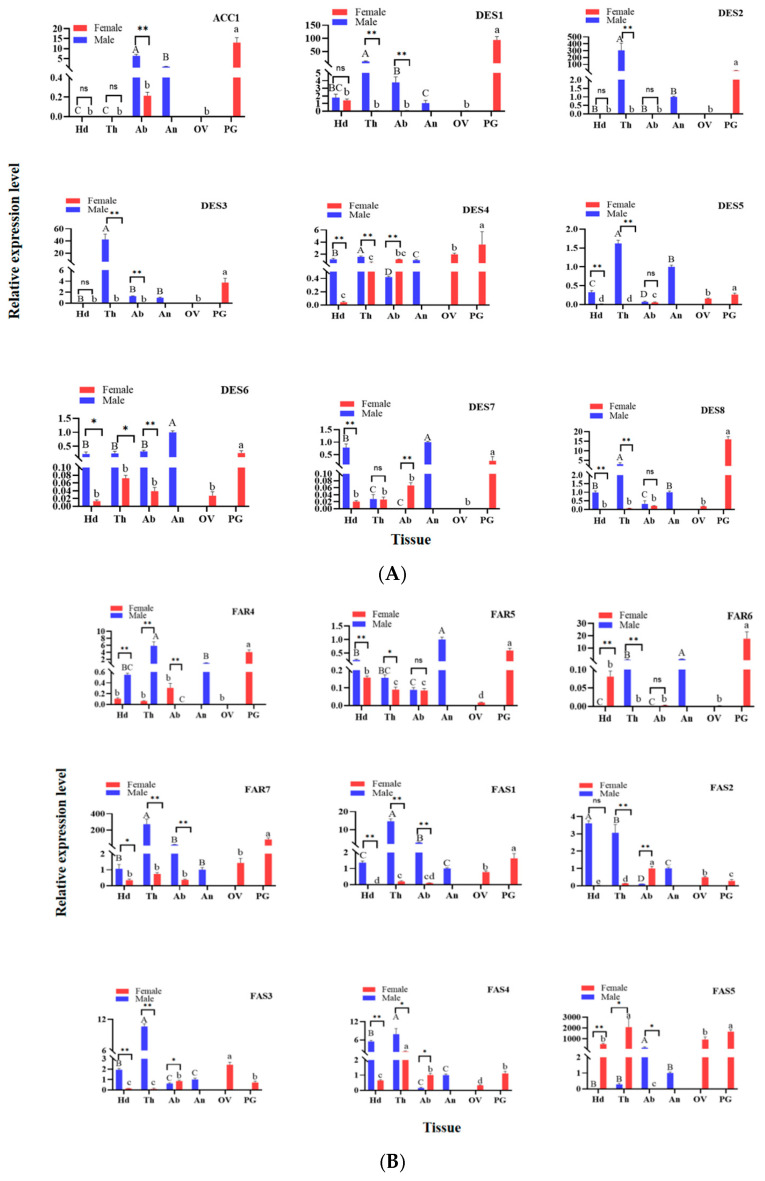

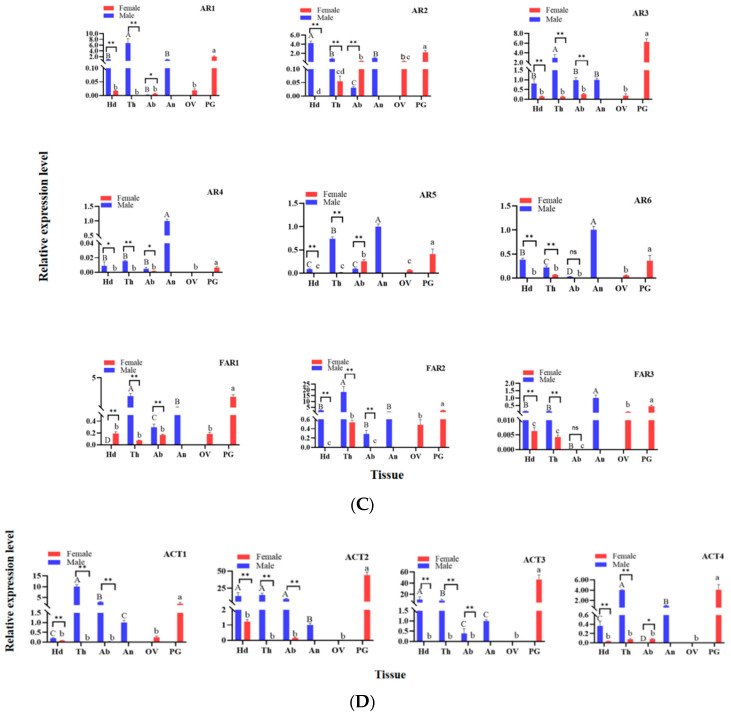
Tissue expression profile analysis of sex pheromone biosynthesis related genes in *G. qinghaiensis.* Male antennae (An); head (with antennae removed) (Hd); thorax (Th); abdomen (Ab); ovary (OV); sex pheromone glands (PG). *RPS15* was used as the internal reference gene to correct the expression level of each tissue, and the expression level of the target gene in the PG was used as a positive control. Error bars indicate the standard error of 3 independent experiments, different uppercase and lowercase letters indicate significant differences between male and female tissues, and single and double asterisks indicate significant differences in the relative expression levels of target genes in the same tissue, and between males and females (*t*-test, * *p* < 0.05; ** *p* < 0.01; ns: not significant). (**A**) ACC1, DES1~8; (**B**) AR1~6, FAR1~3; (**C**) FAR4~7, FAS1~5; (**D**) ACT1~4.

Additionally, *GqinDES6*, *GqinDES7*, *GqinAR4-6*, *GqinFAR3*, and *GqinFAR5* genes were significantly expressed in male adult antennae. Except for *GqinDES4*, *GqinAR2*, *GqinFAR2*, *GqinFAR3*, *GqinFAR7*, *GqinFAS1-5*, and *GqinACT1*, the expression of other genes related to sex pheromone synthesis was relatively high in female ovaries. The expression of other genes was higher in the head, thorax or abdomen, and the expression was different between male and female. (*p* < 0.05) (Figure 3).

## 4. Discussion

For non-model species, transcript sequences can be obtained through de novo assembly by assembling cDNA fragments derived from sequencing. This approach establishes a foundation for various transcriptome studies and other research involving non-model organisms. In this study, a series of bioinformatics analyses were performed, including gene structure annotation, gene expression analysis, and gene function annotation, providing a molecular basis for biological research projects. Transcriptome sequencing was conducted on three samples, resulting in a total of 22.82 Gb of clean data. Each sample yielded clean data of 7.40 Gb or more, with a Q_30_ base percentage of 96.14% or higher. The analysis produced a total of 139,599 transcripts and 56,403 unigenes, with N50 values of 2135 for transcripts and 1757 for unigenes. These results indicate high assembly integrity and ensure the accuracy of the transcriptome analysis [45].

The sex pheromones released by female moths consist of a mixture of components in specific proportions, exhibiting high species specificity. The synthesis of these specific sex pheromone mixtures necessitates the coordinated action of multiple enzymes. Utilizing the gonadal transcriptome data of caterpillars from the *G. qinghaiensis*, this study identified a total of 31 enzyme genes associated with sex pheromone synthesis. Phylogenetic analysis and tissue expression profiling primarily highlighted three genes that are particularly relevant to sex pheromone biosynthesis. These genes represent promising candidates for further functional analysis and may also serve as potential targets for pest control strategies.

In moths, saturated long-chain fatty acids serve as precursors to sex pheromones, with their biosynthesis initiated by acetyl-CoA carboxylase (ACC), which catalyzes the conversion of acetyl-CoA to malonyl-CoA, as the first step in sex pheromone biosynthesis [46]. In the gland of the *G. qinghaiensis*, we identified a transcript encoding ACC. Notably, GqinACC1, a 381 bp coding sequence, exhibited 61.07% amino acid identity with the ACC of *H. assulta* (Protein ID: AKD01721.1). Furthermore, *GqinACC1* was significantly expressed in the female gonads, indicating its crucial role in the acetylation reaction involved in the biosynthesis of sex pheromone fatty acids in *G. qinghaiensis*.

Fatty acid synthase (FAS) catalyzes the conversion of malonyl-CoA and NADPH to produce saturated fatty acids. In the gonadal transcriptome analysis [47,48], we identified five FAS proteins, with lengths ranging from 495 to 783 bp. Notably, the 564 bp coding sequence of FAS4 exhibited 87.23% amino acid identity with the FAS from *S. litura* (Protein ID: XP_022831505.1). Expression profiling revealed that *GqinFAS5* had an extremely high expression level in the gonads, suggesting that this gene may play a significant role in the synthesis of saturated fatty acids in conjunction with malonyl-CoA and NADPH [49].

Specific double bonds are introduced into fatty acid precursors to form fatty acyl-CoA precursors, which are subsequently shortened by β-oxidation to generate various short-chain fatty acyl-CoA precursors. These precursors are further reduced by fatty acyl reductase (FAR) to produce the corresponding alcohols [21,50]. In our analysis of the gonadal transcriptome data, we identified seven FARs homologous to the putative FAR gene. Among these, the 384 bp coding sequence of FAR2 exhibited a high amino acid identity of 96.88% with the FAR from *S. insularis* (Protein ID: QLI61998.1), while the remaining six protein-coding transcripts showed amino acid identities ranging from 64.77% to 81.19% with *H. armigera* and other species. Phylogenetic analysis revealed that GqinFAR6 clustered with pgFARs, and the FAR in this clade, including pgFAR from *B. mori* [50] and SlitFAR3 from *S. litura* [8], exhibited significant expression in pheromone glands. This suggests that *GqinFAR6* may play a crucial role in the synthesis of sex pheromones.

Acyltransferase (ACT) is a key enzyme in the biosynthesis pathway of moth pheromones, facilitating the conversion of fatty acid alcohols into their corresponding esters [51,52]. In this study, we identified a total of four ACT transcripts from related species, including *O. furnacalis*, *D. plexippus*, *T. ni*, and *Spodoptera littoralis*, with amino acid identities ranging from 60% to 75%. The gonad-specific expression of *ACT* in the *Atrijuglans hetaohei* suggests its involvement in the synthesis of corresponding acetates [53]. Notably, *GqinACT3* and *Gqin*ACT4 were specifically expressed in the gonads of female *G. qinghaiensis*, leading to speculation that these two genes also play a role in the synthesis of the corresponding acetates.

Desaturase (DES) enzymes are responsible for introducing double bonds into fatty acyl precursors, a critical step in the biosynthesis of sex pheromones [54]. Previous studies in lepidopteran species have demonstrated the role of Δ11-desaturase in modifying palmitic acid to produce components of sex pheromones [49]. Additionally, Δ9-desaturase converts stearic acid (18:0) to oleic acid and may also convert palmitic acid to palmitoleic acid, although the latter is not involved in the biosynthesis of sex pheromones in *Agrotis ipsilon* [9]. In our analysis, we identified eight DES proteins in the gonads, which exhibited high homology with DES sequences from other lepidopteran insects, ranging from 67.45% to 86.60%. Phylogenetic analysis indicated that GqinDES1, GqinDES6, GqinDES3, GqinDES5, and GqinDES7 clustered within the Δ11-desaturase branch. GqinDES4 was positioned in the Δ9 (16C > 18C) desaturase branch, while GqinDES8 fell into the Δ9 (18C > 16C) desaturase clade. These findings suggest that the sex pheromone biosynthesis pathway in *G. qinghaiensis* may involve desaturation steps mediated by both Δ9 and Δ11 desaturases. Additionally, the expression levels of Gqin*DES1*, Gqin*DES4*, and Gqin*DES8* in the gonads were significantly higher than those in other tissues, suggesting that these three genes may play a crucial role in producing fatty acyl precursors for sex pheromone biosynthesis. They are likely involved in the desaturation step that converts saturated fatty acids (16C) to unsaturated fatty acids by introducing double bonds at the 9th or 11th positions of the carbon chain. In contrast, other *DES* genes did not exhibit gonad-specific expression and may not be involved in sex pheromone desaturation, though they could serve other physiological functions. Further detailed studies are needed to validate this hypothesis [7].

Aldehyde reductase (AR) catalyzes the reduction of aldehyde substrates to their corresponding alcohols in the synthesis of sex pheromones [55]. In our analysis, we identified six transcripts in the gonads of steppe caterpillars that were homologous to the fatty acyl reductase (FAR) of *H. armigera* and *H. assulta*. The proteins encoded by these transcripts exhibited amino acid identities ranging from 58.40% to 80.65% with the homologous FARs (ProteinID: ATJ44537.1, ATJ44541.1, ATJ44498.1, ATJ44502.1, ATJ44539.1, ATJ44505.1). Notably, *GqinAR3* was specifically expressed in the gonads, suggesting its primary role as a reductase in converting sex pheromone components to their corresponding alcohols. In contrast, *GqinAR4-6* were predominantly expressed in the antennae of male adults, indicating their potential involvement in olfactory functions. Additionally, ARs that are highly expressed in other tissues may have roles beyond olfaction [7].

The β-oxidation and fatty acid synthesis pathways involved in moth sex pheromone production are generally similar to those found in the normal metabolism of organisms. Therefore, we would expect no significant differences in the expression of acetyl-CoA carboxylase, fatty acid synthase, and β-oxidation enzyme systems between males and females. However, our experiment revealed notable differences in the expression levels of certain genes between the sexes. This observation leads us to speculate that the sex pheromone synthesis pathway in *G. qinghaiensis* may possess unique characteristics in its β-oxidation and fatty acid synthesis processes. We propose that key genes involved in sex pheromone production—specifically *GqinDES1*, *GqinDES8*, and *GqinFAR6*, which exhibit significant expression in the gonads—should be prioritized for further functional analysis. Additionally, genes that are not specifically expressed in the gonads may be investigated using alternative methodologies.

## Figures and Tables

**Table 1:** Results of transcriptome data from sex pheromone glands of *G. qinghaiensis*.

Sample	Read Sum	BaseSum (Gb)	GC (%)	N (%)	Q_20_ (%)	CycleQ_20_ (%)	Q_30_ (%)
X.1	25,560,576	7.719	39.6	0.01	98.56	100	96.14
X.2	24,505,823	7.400	39.28	0.01	98.67	100	96.42
X.3	25,499,053	7.700	39.64	0.01	98.57	100	96.21
Average	25,188,484	7.607	39.51	0.01	98.60	100	96.26

**Table 2:** Results of the transcriptome assembly from sex pheromone glands of *G. qinghaiensis*.

Length Range	Transcripts	Percentage/%	Unigenes	Percentage/%
300–500	39,928	28.60%	22,694	40.24%
500–1000	41,164	29.49%	16,624	29.47%
1000–2000	30,922	22.15%	9273	16.44%
2000+	27,585	19.76%	7812	13.85%
Total	139,599		56,403	

**Table 3:** Statistics of gene functional annotation for transcriptomes from sex pheromone glands of *G. qinghaiensis*.

Anno_Database	Annotated_Number	Percentage/%	300 ≤ Length < 1000	Length ≥ 1000
COG_Annotation	4865	8.6	935	3930
GO_Annotation	10,228	18.13	2691	7537
KEGG_Annotation	20,576	36.48	8824	11,752
KOG_Annotation	10,721	19.01	2826	7895
Pfam_Annotation	11,603	20.57	2787	8816
Swissprot_Annotation	11,001	19.50	2919	8082
NR_Annotation	13,781	24.43	4000	9781
All_Annotated	20,729	36.75	8941	11,788

Note: 300 ≤ Length < 1000-, indicates the number of Unigene notes to the database with a length between 300 and 1000 bases; Length ≥ 1000-, indicates the number of Unigene comments with a length of more than 1000 bases to the database.

**Table 4:** Bioinformatics analysis of sex pheromone biosynthesis related genes in *G. qinghaiensis*.

Gene	Nucleotide	Transmembrane Domains	CDS	Amino Acid	Blastp Test and Verify			
	Length/bp		Integrity	Length/aa	Species	GenBank Accession	E-Value	Identity/%
*ACC1*	381	1	No	126	*Helicoverpa assulta*	AKD01721.1	2 × 10^−41^	61.07%
*DES1*	990	5	Yes	329	*S. exigua*	ARD71185.1	0	74.61%
*DES2*	966	4	Yes	321	*S. exigua*	ARD71182.1	0	86.60%
*DES3*	1098	5	Yes	365	*Grapholita molesta*	AUC64291.1	1 × 10^−147^	67.45%
*DES4*	993	4	Yes	330	*S. insularis*	QLI61972.1	3 × 10^−161^	69.13%
*DES5*	1368	5	Yes	455	*Agrotis segetum*	AID66662.1	0	76.77%
*DES6*	939	2	Yes	312	*Danaus plexippus plexippus*	OWR48667.1	3 × 10^−152^	70.67%
*DES7*	1116	4	Yes	371	*Spodoptera littoralis*	AAQ74260.1	0	79.40%
*DES8*	1065	4	Yes	354	*Sesamia inferens*	AII21941.1	0	82.77%
*AR1*	1023	0	Yes	340	*H. assulta*	ATJ44537.1	0	77.35%
*AR2*	1014	0	Yes	337	*H. assulta*	ATJ44541.1	0	71.77%
*AR3*	504	0	Yes	167	*H. assulta*	ATJ44498.1	2 × 10^−81^	68.86%
*AR4*	801	0	Yes	266	*Helicoverpa armigera*	ATJ44502.1	2 × 10^−129^	59.63%
*AR5*	927	0	Yes	308	*H. assulta*	ATJ44539.1	0	80.65%
*AR6*	1167	0	Yes	388	*H. armigera*	ATJ44505.1	3 × 10^−165^	58.40%
*FAR1*	1557	2	Yes	518	*Pectinophora gossypiella*	XP_049883830.1	0	68.42%
*FAR2*	384	0	NO	127	*S. insularis*	QLI61998.1	9 × 10^−83^	96.88%
*FAR3*	1602	2	Yes	533	*H. armigera*	AKD01766.1	0	78.30%
*FAR4*	1395	0	Yes	464	*H. assulta*	AKD01789.1	0	64.77%
*FAR5*	1566	1	Yes	521	*H. armigera*	AKD01770.1	0	76.06%
*FAR6*	705	0	Yes	234	*H. armigera*	AKD01771.1	2 × 10^−120^	81.19%
*FAR7*	1578	2	Yes	525	*Helicoverpa zea*	XP_047038839.1	0	80.04%
*FAS1*	495	0	Yes	164	*H. armigera*	XP_049700707.1	3 × 10^−82^	77.44%
*FAS2*	744	0	Yes	247	*Dioryctria abietella*	QZC92075.1	1 × 10^−123^	69.35%
*FAS3*	783	0	Yes	260	*Spodoptera frugiperda*	XP_050550085.1	3 × 10^−120^	68.58%
*FAS4*	564	0	Yes	187	*S. litura*	XP_022831505.1	2 × 10^−107^	87.23%
*FAS5*	519	0	Yes	172	*H. armigera*	XP_049699195.1	2 × 10^−71^	67.82%
*ACT1*	531	0	Yes	176	*Ostrinia furnacalis*	XP_028157379.1	2 × 10^−91^	74.12%
*ACT2*	732	0	Yes	243	*D. p. plexippus*	OWR41901.1	1 × 10^−93^	63.51%
*ACT3*	783	0	Yes	260	*Trichoplusia ni*	XP_026730350.1	3 × 10^−137^	73.46%
*ACT4*	1410	0	Yes	469	*Spodoptera littoralis*	CAB3515140.1	0	69.79%

**Table 5:** Physicochemical properties of sex pheromone biosynthesis related proteins in *G. qinghaiensis*.

Protein	Amino Acids	Molecular Weight/KD	Isoelectricpoint/PI	Negatively Charged Residues	Positively Charged Residues	Instability Index	Aliphatic Index	Average Hydropathicity
ACC1	127	14.05	4.96	20	13	55.12	63.78	−0.613
DES1	329	37.56	6.89	31	30	40.27	104.62	0.141
DES2	321	37.28	8.93	26	31	39.14	97.73	0.117
DES3	365	41.77	9.3	25	37	37.3	84.88	−0.095
DES4	330	38.55	9.04	27	34	30.02	89.27	−0.111
DES5	455	53.34	8.65	42	47	39.09	89.82	−0.164
DES6	312	36.14	8.77	28	32	44.41	81.92	−0.241
DES7	371	42.78	6.43	40	36	36.49	94.07	−0.091
DES8	354	40.95	8.81	33	37	34.52	87.34	−0.149
AR1	340	39.12	6.55	44	43	36.43	88.24	−0.389
AR2	337	37.83	5.77	40	35	36.42	96.26	−0.166
AR3	167	19.06	9.23	18	23	29.46	92.75	−0.249
AR4	266	30.12	5.86	31	28	33.34	97.48	−0.161
AR5	309	34.98	8.78	35	40	24.63	89.58	−0.286
AR6	388	43.87	8.37	43	46	40.27	97.99	−0.147
FAR1	518	59.15	7.87	61	63	35.18	101.62	−0.069
FAR2	128	14.03	5.1	16	11	21.41	101.17	0.022
FAR3	533	60.50	6.24	63	60	27.81	94.93	−0.082
FAR4	464	53.11	8.99	51	60	33.01	90.32	−0.138
FAR5	521	59.30	8.82	55	62	40.43	107.52	−0.052
FAR6	234	27.17	9.43	22	30	42.35	100.73	−0.024
FAR7	525	59.44	8.53	59	64	36.56	99.9	0.025
FAS1	165	18.91	5.29	25	16	45.17	86.79	−0.339
FAS2	248	27.39	6.05	26	21	44.06	100.28	0.025
FAS3	261	29.17	7.08	31	31	31.03	90.04	−0.324
FAS4	188	20.50	8.52	20	22	31.73	107.34	0.128
FAS5	173	18.77	6.41	17	16	38.05	95.95	0.042
ACT1	176	19.93	5.81	26	24	38.92	65.97	−0.553
ACT2	243	27.43	4.71	37	25	42.17	77.45	−0.322
ACT3	260	29.41	6.61	36	35	20.31	83.35	−0.361
ACT4	469	51.87	8.75	50	57	31.14	84.22	−0.359

## Data Availability

The original contributions presented in the study are included in the article/Supplementary Materials; further inquiry can be directed to the corresponding authors.

## Supplementary Materials

The following supporting information can be downloaded at: https://www.mdpi.com/article/10.3390/genes16091046/s1, Table S1: Primers used in the study.
